# Diabetes Prevalence in Sweden at Present and Projections for Year 2050

**DOI:** 10.1371/journal.pone.0143084

**Published:** 2015-11-30

**Authors:** Tomas Andersson, Anders Ahlbom, Sofia Carlsson

**Affiliations:** 1 Institute of Environmental Medicine, Karolinska Institutet, Stockholm, Sweden; 2 Center for Occupational and Environmental Medicine, Stockholm County Council, Stockholm, Sweden; Heinrich-Heine University, Faculty of Medicine, GERMANY

## Abstract

**Background:**

Data on the future diabetes burden in Scandinavia is limited. Our aim was to project the future burden of diabetes in Sweden by modelling data on incidence, prevalence, mortality, and demographic factors.

**Method:**

To project the future burden of diabetes we used information on the prevalence of diabetes from the national drug prescription registry (adults ≥20 years), previously published data on relative mortality in people with diabetes, and population demographics and projections from Statistics Sweden. Alternative scenarios were created based on different assumptions regarding the future incidence of diabetes.

**Results:**

Between 2007 and 2013 the prevalence of diabetes rose from 5.8 to 6.8% in Sweden but incidence remained constant at 4.4 per 1000 (2013). With constant incidence and continued improvement in relative survival, prevalence will increase to 10.4% by year 2050 and the number of afflicted individuals will increase to 940 000. Of this rise, 30% is accounted for by changes in the age structure of the population and 14% by improved relative survival in people with diabetes. A hypothesized 1% annual rise in incidence will result in a prevalence of 12.6% and 1 136 000 cases. Even with decreasing incidence at 1% per year, prevalence of diabetes will continue to increase.

**Conclusion:**

We can expect diabetes prevalence to rise substantially in Sweden over the next 35 years as a result of demographic changes and improved survival among people with diabetes. A dramatic reduction in incidence is required to prevent this development.

## Introduction

Diabetes is increasing world wide and this development is expected to continue: In Europe, diabetes prevalence has been projected to rise from 8.5% (2013) to 10.3% (2035) in adults 20–79 years, as a consequence of the aging population [1]. However, if incidence of diabetes is rising as well, this forecast is an underestimation. Changes in survival in patients with diabetes will also affect the future burden of diabetes and according to data from the US [2], UK [3], Denmark [4] and Sweden [5–6] mortality is decreasing faster in people with diabetes than in the general population. The influence of changes in incidence and survival may be substantial. In the US, Boyle et al modeled the prevalence of diabetes to be 21% by 2050 in the light of stable incidence and high mortality, whereas a rise in incidence and relatively low mortality will result in prevalence of 33% [7]. As far as we know, only one previous study [8]accounted for trends in incidence and survival while forecasting the future burden of diabetes in Europe.

Prevalence of diabetes is reportedly lower in Scandinavian Countries than in other parts of Europe [1] and the US [9], but recent studies suggest that it has been rising since the 1990:ies. Data on the future burden of diabetes in Scandinavia is limited to the projections from the International Diabetes Federation (IDF) [1], where no information on trends in mortality or incidence is included. Against this background our aim was to project the future burden of diabetes in Sweden by modelling data on prevalence, incidence, and survival in patients with diabetes together with population projections, taking into account changes in survival and demographic structure of the general population.

## Material and Methods

### Diabetes

The national drug prescription registry records all dispensed prescription drugs for the entire Swedish population since 1 July 2005 [10–11]. This registry can hence be expected to cover all individuals with pharmacologically treated diabetes in Sweden [12]. The drugs are classified according to the Anatomical Therapeutic Chemical (ATC) classification system [13] and those belonging to ATC group 10 (insulin and oral antidiabetic drugs) where used in this study. No distinction was made between different forms of diabetes. This was used to calculate the prevalence of pharmacologically treated diabetes during the period 2007–2013. To account for non-pharmacologically treated diabetes, which accounted for 22.6% of all diabetes in adults in Sweden in 2013, according to recently published data from the national diabetes registry (NDR) [14], we amplified the prescription registry-based diabetes count by a factor of 1.292.

### Mortality

Age-specific death risks of the general population 2007–2013, was based on official population statistics produced by Statistics Sweden. Information on the age- specific, relative excess mortality in people with diabetes (irrespective of diabetes type) came from previously published findings from the Stockholm County for the period 1994–2011. This study included 63 616 randomly selected individuals of the adult (≥18 years) population of Stockholm County (~2.1 million inhabitants representing 1/5 of the Swedish population) who participated in a survey in 1994, 2002, 2006, or 2010. Through linkage to the national death registry we observed 2389 deaths over the period 1994–2011, and estimated that the relative excess mortality in people with diabetes declined annually by 1.6% [6].

### Population forecast

Statistics Sweden provides annual forecasts of the population of Sweden based on assumptions regarding age and sex specific death rates, fertility rates, and migration. We used data from its latest projection for the period 2014–2050 [15]

### Ethics Statement

The study was based on publicly available data and previously published data and no individual data was used, hence, no ethical approval or consent was warranted.

### Statistical methods

Because diabetes is a chronic disease there is in a closed cohort a straightforward relation between incidence, prevalence, and survival: The prevalence at the end of one year is the prevalence one year earlier minus the deaths during that year plus the incident cases during the same year. Thus, the prevalence at the end of a year for a specific birth cohort and gender was calculated as the sum of two parts: First the prevalence one year earlier was multiplied with the chance of survival for an individual with diabetes. Second the incident cases were calculated by multiplying the cumulative incidence of diabetes with the prevalence of non diabetes at the end of the previous year. The prevalence of non-diabetes was taken as the total population at that time minus the surviving individuals with diabetes from one year earlier. That is:

$$p_{y}=\frac{\left(1-m_{1,y-1}\right)p_{y-1}+\left(1-m_{y-1}-\left(1-m_{1,y-1}\right)p_{y-1}\right)i_{y}}{1-m_{y-1}}$$ (1)

where:

p_y_ = prevalence of diabetes at the end of year y for a specific birth cohort and gender

m_y_ = death risk during year y for a specific birth cohort and gender in the total population

m_1,y_ = death risk during year y for a specific birth cohort and gender among all individuals with diabetes

i_y_ = cumulative incidence (risk) of diabetes during year y for a specific birth cohort and gender

During the base line period of 2007–2013, the prevalence of pharmaceutically treated diabetes was known from the prescription registry (amplified to account for non-pharmacologically treated diabetes) and mortality for the general population was known from statistics Sweden. The excess mortality among individuals with diabetes was known from our previous study [6]. Thus, Eq 1 can be solved for the cumulative incidence:

$$i_{y}=\frac{\left(1-m_{y-1}\right)p_{y}-\left(1-m_{1,y-1}\right)p_{y-1}}{1-m_{y-1}-\left(1-m_{1,y-1}\right)p_{y-1}}$$ (2)

Eq 2 was applied separately for birth cohorts and genders for each of the years in the base line period 2007–2013, thus providing the cumulative incidences. For the forecasting period from 2014–2050,formula (1) was used to calculate the prevalence in a yearly step by step fashion. Population figures and death risks were then taken from statistics Sweden's forecasts while different scenarios were applied regarding the future incidence of diabetes and also regarding the future excess mortality among people with diabetes.

Since the primary data for prevalence and incidence calculations were counts taken from public registries they were not designed primarily for use in epidemiologic research and the measures that we were using had to be adopted accordingly. Thus, prevalence in our calculations was the number of persons with dispensed prescriptions during the year divided by the number of people at the start of that year and death risks among all individuals with diabetes were calculated as the expected proportion of deceased among people that had collected diabetes medication at least once.

The estimates of prevalence and incidence does only rely on proportions and thus would be unaffected by changes in population size. This makes it possible to apply our method to the actual Swedish population although it is not a closed cohort.

### Projections

We projected the future prevalence during the period 2014–2050, in relative and absolute numbers. Four scenarios (displayed in Table 1) were modelled with different assumptions regarding the future incidence and mortality; In Scenario A we assume that the relative risk of death for those with diabetes continues to decrease by 1.6% per year and assume that age specific incidence is constant at the levels observed in 2013 (summarized in S1 Table). In scenario B) the relative risk of death associated with diabetes decreases as in scenario A and incidence of diabetes increases by 1% annually, with the estimated age-specific incidence in 2013 as starting points. In scenario C) mortality decreases as in scenario A and B but age-specific incidence decreases at 1% annually. Scenario D implies that mortality in people with diabetes improves at the same rate as in people without diabetes and incidence is constant. Based on these scenarios we estimated prevalence and number of people with diabetes 2014–2050.

**Table 1:** Projected prevalence and number of cases of diabetes 2014–2050.

	Incidence 2014–2050	Relative diabetes mortality risk 2014–2050	Prevalence in 2050	No. patients in 2050
**Scenario A. Incidence is constant, mortality decreases**	Constant at levels observed in 2013	Continues to decrease by 1.6% annually	10.4% (all), 8.6% (women), 12.2% (men)	940 000 (all), 387 000 (women), 553 000 (men)
**Scenario B. Incidence increases, mortality decreases**	1% annual increase	Continues to decrease by 1.6% annually	12.6% (all), 10.4% (women), 14.8% (men)	1 136 000 (all), 467 000 (women), 669 000 (men)
**Scenario C. Incidence decreases, and mortality decreases**	1% annual decrease	Continues to decrease by 1.6% annually	8.7% (all), 7.2% (women), 10.1% (men)	783 000 (all), 324 000 (women), 459 000 (men)
**Scenario D. Incidence is constant, mortality is constant**	Constant at levels observed in 2013	Relative risk is constant at levels observed in 2013	9.7% (all), 8.0% (women), 11.4% (men)	880 000 (all), 361 000 (women), 518 000 (men)

## Results

### Prevalence and Incidence of diabetes 2007–2013 based on the prescription registry

Prevalence increased from 5.8 to 6.8% in Sweden between 2007 and 2013; from 6.6 to 7.9% in men and from 5.1 to 5.8% in women (Table 2). The rise occurred across all age categories but was most pronounced in people aged 65 or older (S1 Fig). As expected, prevalence increased with age; in 2013, 12.8% of women and 18.8% of men ≥65 years had diabetes. Incidence remained relatively stable during the period 2007–2013 and was estimated at 4.4 (95% CI; 4.1–4.9) per 1000 in 2013 (5.5, 95% CI; 5.2–6.1 in men and 3.3, 95% CI; 3.0–3.8 in women) (S1 Table).

**Table 2:** Prevalence of diabetes in Sweden 2007 to 2013 by age and sex.

	Women				Men				Total			
	20–44	45–64	≥65	≥20 years	20–44	45–64	≥65	≥20 years	20–44	45–64	≥65	≥20 years
**2007**	1.2%	4.6%	11.7%	5.1%	1.5%	7.4%	16.0%	6.6%	1.4%	6.0%	13.6%	5.8%
**2008**	1.3%	4.8%	12.0%	5.2%	1.5%	7.7%	16.5%	6.9%	1.4%	6.2%	14.0%	6.1%
**2009**	1.3%	4.9%	12.1%	5.3%	1.5%	7.8%	17.0%	7.1%	1.4%	6.4%	14.3%	6.2%
**2010**	1.3%	5.0%	12.4%	5.5%	1.6%	8.0%	17.6%	7.4%	1.4%	6.5%	14.7%	6.4%
**2011**	1.3%	5.1%	12.5%	5.6%	1.6%	8.1%	18.0%	7.6%	1.4%	6.6%	15.0%	6.6%
**2012**	1.3%	5.2%	12.7%	5.7%	1.6%	8.2%	18.5%	7.8%	1.5%	6.7%	15.3%	6.7%
**2013**	1.4%	5.2%	12.8%	5.8%	1.6%	8.3%	18.8%	7.9%	1.5%	6.8%	15.6%	6.8%

### Projected prevalence and number of people with diabetes 2014–2050

**Fig 1:**
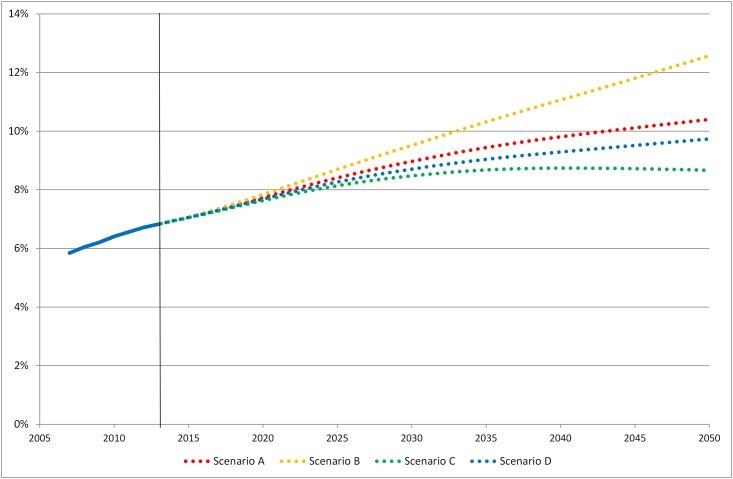
Prevalence of diabetes in Sweden 2007–2013 and projections for 2014–2050.

**Fig 2:**
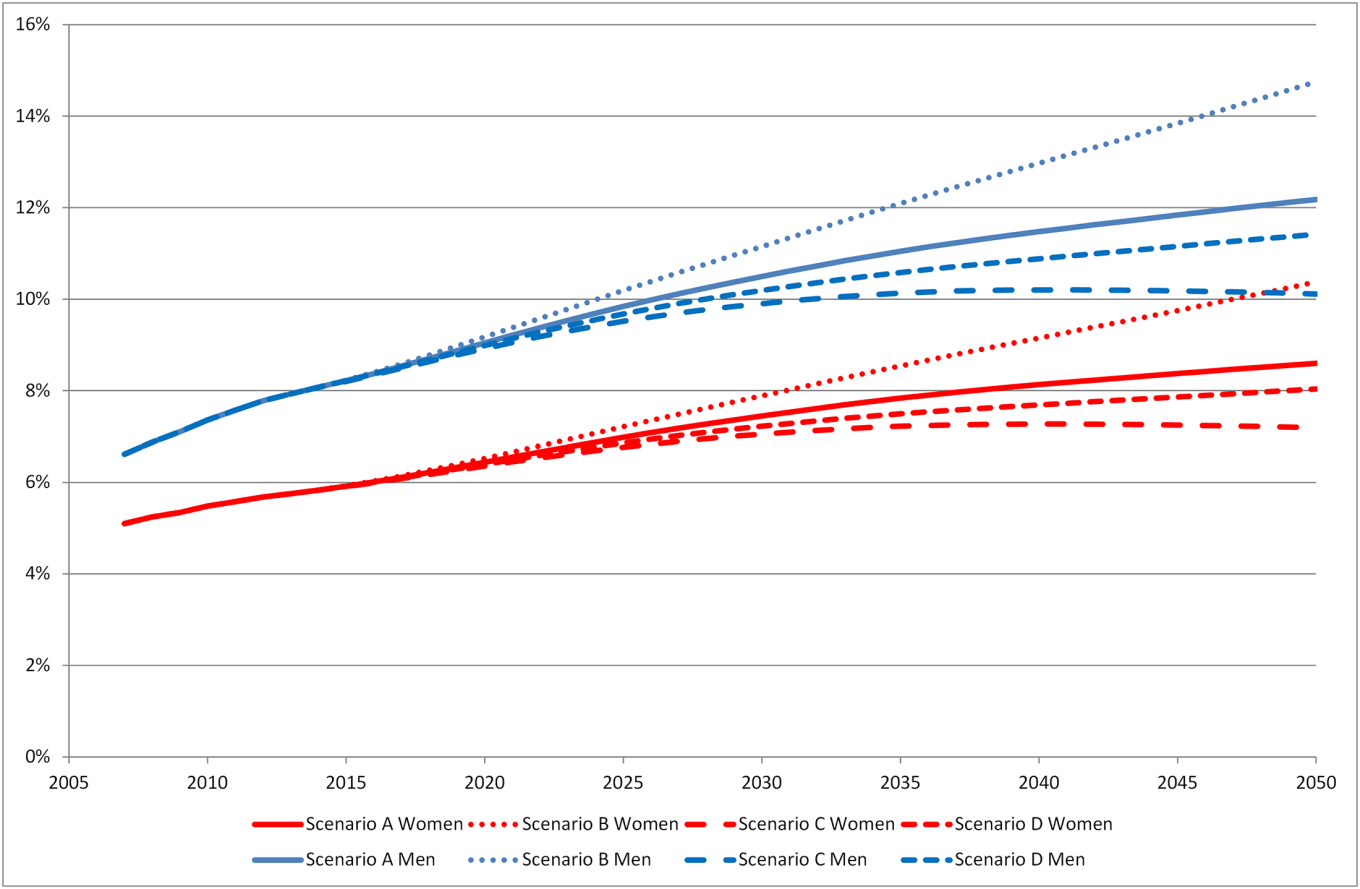
Prevalence of diabetes in Sweden 2007–2013 and projections for 2014–2050, by sex.

**Fig 3:**
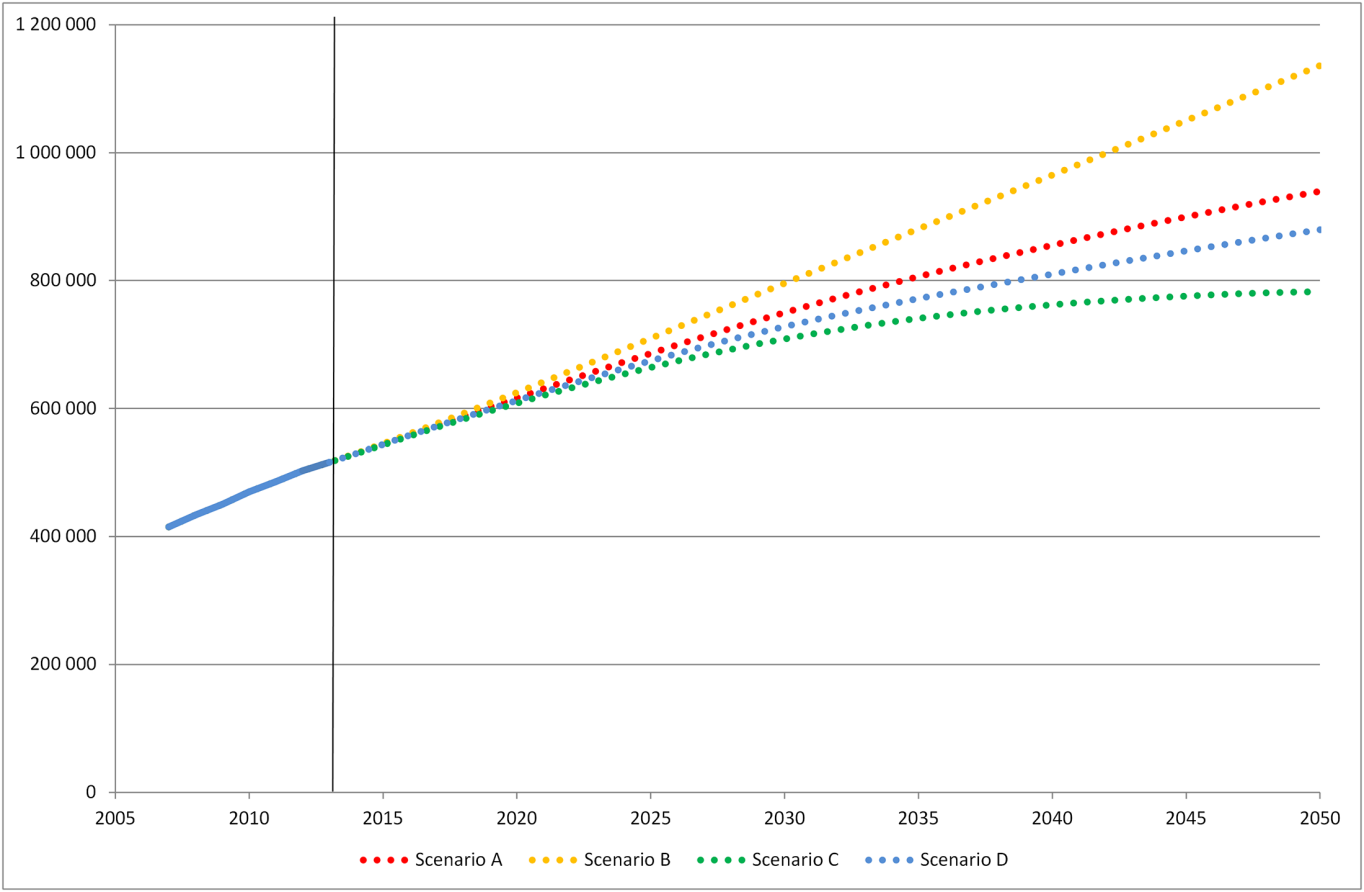
Number of patients with diabetes in Sweden 2007–2013 and projections for 2014–2050.

**Scenario A:** With a continued relative decline in mortality risk in people with diabetes and constant incidence, the prevalence of diabetes is projected to increase in Sweden and reach 10.4% by year 2050 (Fig 1); 8.6% in women and 12.2% in men (Fig 2). The number of people with diabetes will also increase and by year 2050, 940 000 inhabitants will have diabetes (Fig 3). Most of the rise in prevalence will occur at age 65 and above (S1 Fig). According to our projection, there will be a shift in the age distribution of the diabetes patients; in 2013, 59% of all individuals with diabetes were 65 years or older compared to 69% in 2050. **Scenario B:** If mortality risk continues to decline faster in people with diabetes than in those without as in Scenario A, but incidence starts to rise at 1% per year, we can expect diabetes prevalence of 12.6% in 2050 (10.4% in women and 14.8% in men) (Fig 1). With this scenario 1 136 000 inhabitants will have diabetes in 2050 (Fig 3). **Scenario C:** In the third scenario, mortality in people with diabetes decline as in Scenario A and B but incidence decreases at 1% per year. This would lead to prevalence of 8.7% by year 2050 and 783 000 individuals with diabetes. **Scenario D:** If mortality risk in people with diabetes stabilizes and incidence is constant, prevalence would reach 9.7% and the number of afflicted individuals would be 880 000 by year 2050.

### Relative importance of factors affecting the future number of patients

In scenario A with stable incidence and decreasing relative mortality, the number of cases of diabetes will increase by 182% between 2013 and 2050. Of this increase, 36% is accounted for by changes in population size and 30% by an upward shift in in the age distribution of the population, and 14% to improved relative survival in people with diabetes. Besides this, falling mortality rates in the general population also contribute to increasing number of people with diabetes. The influence of these factors is not mutually exclusive since falling mortality rates influence age structure as well as population size.

## Discussion

Our findings indicate that if the current trends continue, prevalence of diabetes will rise in Sweden from 6.8% (2013) to 10.4% by year 2050, with 940 000 affected inhabitants. The primary driving forces behind this development are changes in age structure and population size, together with mortality that decrease faster in people with diabetes than in those without.

The observed prevalence of diabetes 2007–2013 was higher than in previous Swedish reports [6, 16–18], but this is on the other hand the first time that Swedish diabetes prevalence has been presented based on registry data, with complete coverage for the whole population. These results challenge the view that diabetes is less frequent in Sweden than in other parts of Europe [1]. Our projections also indicate that in the future, we can expect a similar rise in diabetes prevalence as forecasted in the UK; where Holman et al. [19] projected that diabetes prevalence would rise to 9.5% in 2030, and Germany; where a prevalence of 9.4% by year 2040 was projected [20]. In contrast, the IDF forecast suggests that diabetes prevalence will be stable in Sweden until 2035 [1]. Notably, their projection is standardized for age which masks a rise due to changes in the age structure, and furthermore, no assumptions were made regarding in changes in incidence or mortality.

Prevalence of diabetes in Sweden rose between 2007 and 2013, but incidence remained relatively constant. This fits with our previous observations from the Stockholm County where incidence of diabetes rose by 3% annually between 1990 and 2002, but leveled off from 2002 onwards. A similar development has also been reported in Denmark [4]. If incidence of diabetes would start to rise even at a modest level, the influence on the future burden of diabetes would be substantial; a 1% annual increase in incidence would generate a prevalence of 12.6% by year 2050 and the number of patients would be 21% higher than in a scenario with constant incidence. This scenario is not unlikely given that prevalence of overweight and obesity has been increasing steadily in Sweden over the last decades [6, 16]. Most importantly, even with a substantial decline in incidence, prevalence of diabetes will continue to increase in Sweden as a result of changes in age structure and survival.

A rise in the number of diabetes patients will have a major impact on public health since diabetes is afflicted with a range of co-morbidities including an increased risk of cardiovascular disease and premature death [21–22]. It will also have consequences for public spending and planning of health care services. Data on the economic consequences of diabetes in Sweden is scarce. However, according to the study by Waldeyer et al. a rise in prevalence from 5.7 to 9.4% in Germany will increase the annual medical costs for diabetes by 85% [20]. According to our calculations a 2.3% annual reduction in incidence is required to counterbalance the rise in prevalence that is expected to occur due to improved survival and the upward shift in the age distribution of the population. Obesity is by far the most important modifiable risk factor for type 2 diabetes, increasing the risk 7-fold [23]. In order to accomplish a substantial reduction in diabetes incidence there is a need for effective prevention and a significant reduction in levels of overweight and obesity.

### Limitations

Strengths of these projections include the use of registry data for assessment of diabetes prevalence, differential mortality in people with and without diabetes together with official population projections. The Swedish drug prescription registry covers all dispensed prescriptions from Swedish pharmacies [10] and can thus be expected to include all pharmacologically treated patients with diabetes. Patients only treated by diet are missed and to account for this we used information from the Swedish Diabetes Registry on treatment regiments in Swedish patients [15]. In 2013, 23% of Swedish diabetes patients were treated by diet only. It is possible that the proportion of patients with different kinds of treatment will change in the future, but this will not bias our projections since the sum of patients would remain the same. No distinction could be made between different forms of diabetes. Notably, Sweden has the second highest incidence of type 1 diabetes, but type 2 diabetes still accounts for 85–90% of all diabetes in adults [15] and hence, the results primarily pertain to this diabetes form. Undiagnosed diabetes was not included. The IDF includes undiagnosed diabetes at a proportion of 36.6% in their projections [1]. Adding this number to our estimations (scenario A) indicates that 12.3% of the Swedish population will have diabetes by year 2050. In our projections we assumed that the relative mortality in people with diabetes will continue to improve faster in people with diabetes than in the general population, and the rate of improvement was based on observations from Stockholm County for the period 1994–2011 [6]. A similar trend has also been reported from other parts of Europe [3–4] and the US [2]. However, if the rate of improvement is higher in Stockholm compared to the country as a whole we would overestimate the future burden of diabetes. Notably, if survival in people with diabetes would stabilize and follow the same pattern as in the general population diabetes prevalence would increase until year 2030 and then level off. Most previous attempts to project diabetes did not take into account changes in relative mortality [1, 18–19], and this will most likely lead to an underestimation of the future prevalence. One consideration is whether the relative diabetes mortality observed in Stockholm County is generalizable to the population of Sweden as a whole. Still, as long as the difference in mortality between people with and without diabetes is similar in Stockholm as in the rest of Sweden, this generalization should hold. Finally, immigration of people with higher (or lower) diabetes risk may influence the future incidence of diabetes in ways that are difficult to foresee at present.

### Conclusion

Public health is improving in many areas and life expectancy is increasing in the general population and even more so among people with diabetes. Paradoxically, this positive development will have a negative effect on the future burden of diabetes in Sweden; the prevalence and number of cases will increase substantially over the next 35 years, even if the risk of developing diabetes per se would remain constant or even decrease. To reduce the future prevalence of diabetes, a substantial reduction in incidence is required. The health care system needs to be prepared to meet the needs of this growing number of patients with diabetes.

## Supporting Information

S1 Fig Prevalence of diabetes in Sweden 2007–2013 and projections for 2014–2050 by age (Scenario A). (TIF)

S1 Table Approximated incidence of diabetes (per 1000) in Sweden 2007 to 2013 by age and sex. (DOCX)
