# Supplementary figures and images for: Diabetes Prevalence in Sweden at Present and Projections for Year 2050
Source: PLoS One. 2015 Nov 30;10(11):e0143084. doi: 10.1371/journal.pone.0143084 (PMC4664416; doi:10.1371/journal.pone.0143084)

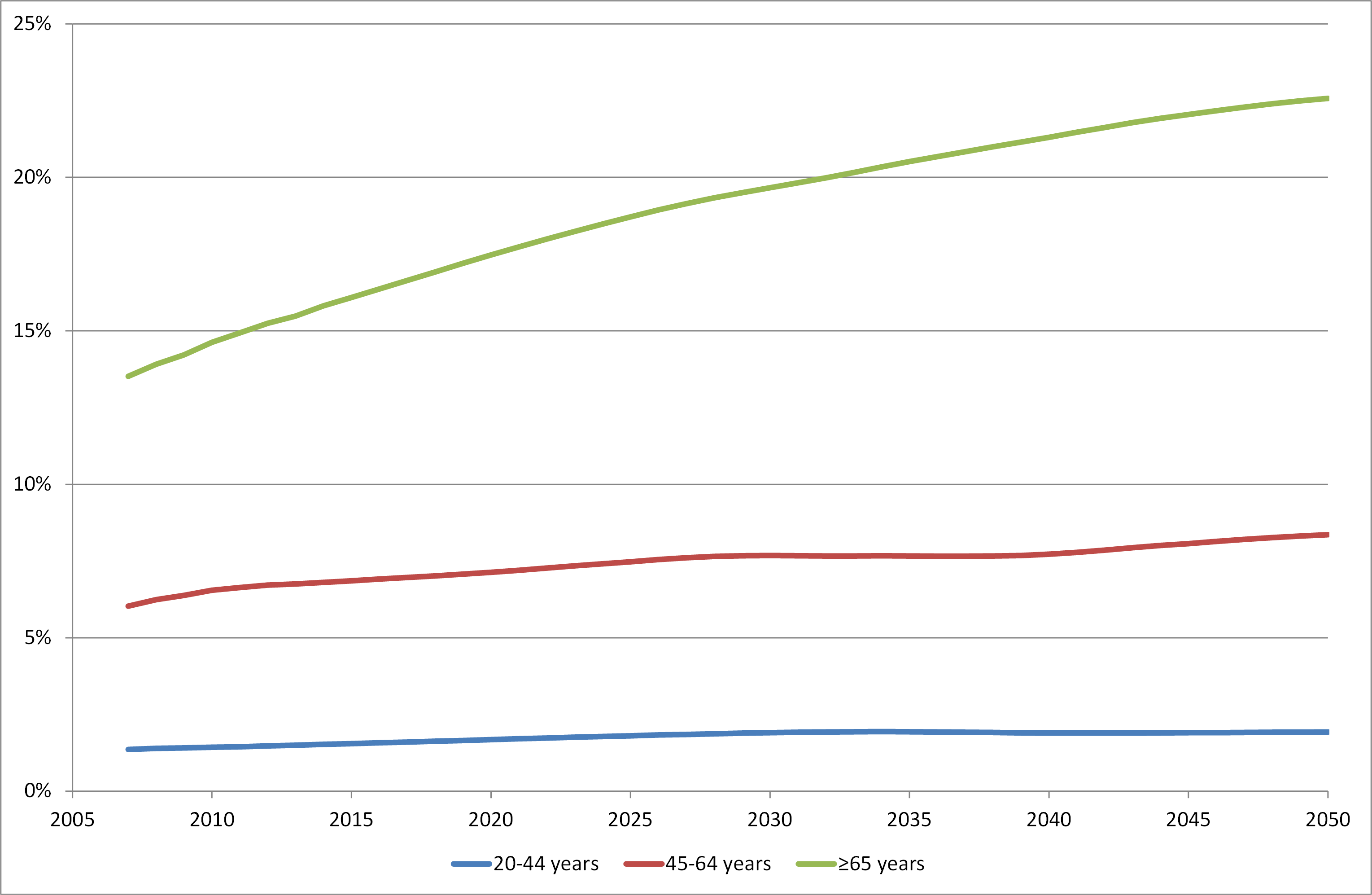

Supplement: S1 Fig — (TIF) [file pone.0143084.s001.tif]
