# Supplementary material for: Diabetes Prevalence in Sweden at Present and Projections for Year 2050
Source: PLoS One. 2015 Nov 30;10(11):e0143084. doi: 10.1371/journal.pone.0143084 (PMC4664416; doi:10.1371/journal.pone.0143084)
Supplement: S1 Table — (DOCX) [file pone.0143084.s002.docx]

##### S1 Table. Approximated incidence of diabetes (per 1000) in Sweden 2007 to 2013 by age and sex.

|  | **Women**  **20-44** | **45-64** | **≥65** | **≥20 years** |  | **Men**  **20-44** | **45-64** | **≥65** | **≥20 years** |  | **Total**  **20-44** | **45-64** | **≥65** | **≥20 years** |
| --- | --- | --- | --- | --- | --- | --- | --- | --- | --- | --- | --- | --- | --- | --- |
| **2007** | 0.6 | 4.6 | 7.4 | 3.7 |  | 1.3 | 7.9 | 12.5 | 6.0 |  | 1.0 | 6.3 | 9.7 | 4.8 |
| **2008** | 0.8 | 5.1 | 8.4 | 4.2 |  | 1.4 | 8.8 | 13.5 | 6.6 |  | 1.1 | 7.0 | 10.7 | 5.4 |
| **2009** | 0.6 | 4.7 | 7.1 | 3.7 |  | 1.4 | 8.6 | 12.6 | 6.3 |  | 1.0 | 6.7 | 9.6 | 5.0 |
| **2010** | 0.8 | 5.3 | 7.9 | 4.2 |  | 1.4 | 8.9 | 13.8 | 6.7 |  | 1.1 | 7.1 | 10.6 | 5.4 |
| **2011** | 0.7 | 4.7 | 6.6 | 3.6 |  | 1.4 | 8.2 | 12.4 | 6.2 |  | 1.1 | 6.5 | 9.2 | 4.9 |
| **2012** | 0.8 | 4.8 | 6.6 | 3.7 |  | 1.4 | 8.2 | 12.1 | 6.2 |  | 1.1 | 6.5 | 9.1 | 4.9 |
| **2013** | 0.8 | 4.4 | 5.8 | 3.3 |  | 1.3 | 7.5 | 10.3 | 5.5 |  | 1.1 | 6.0 | 7.8 | 4.4 |
